# A 13-year longitudinal study of students who enter kindergarten as English learners: early vs. late reclassified fluent English learners

**DOI:** 10.3389/fpsyg.2025.1562699

**Published:** 2025-08-29

**Authors:** Amado M. Padilla, Xinjie Chen, Elizabeth M. Swanson, Diana Mercado-Garcia

**Affiliations:** ^1^Stanford University, Stanford, CA, United States; ^2^The Chinese University of Hong Kong, Hong Kong (SAR), China; ^3^University of Maryland, College Park, MD, United States; ^4^California Education Partners, San Francisco, CA, United States

**Keywords:** English learners, reclassification, early-RFEP, academic achievement, language outcomes, parent education, gender

## Abstract

Research on Long Term English Learners (LTEL), especially their negative academic outcomes is extensive, but Early Reclassified Fluent English Proficient (E-RFEP) students and their potential benefits are largely underexplored. This study analyzed longitudinal data for 13 years (kindergarten through 12th grade) from 1,152 students classified as English Learner (ELs) when they entered kindergarten in three elementary school (K-8) districts, tracking their academic outcomes through middle and high school. Findings show: (1) significant variation in reclassification timing; (2) demographic and socioeconomic factors were significant predictors of E-RFEP, including parent education, ethnicity, and gender; (3) E-RFEP contributed to higher academic achievement compared to LTEL peers and a comparison group of English only (EO) students. This work contributed to balancing the heavy focus on the languishing of LTELs and highlighted the flourishing of E-RFEP. Findings provided evidence-based implications for educators and policy makers on the current assessment and instructional practices for reclassification, as well as needed support for LTEL students to prosper academically.

## Introduction

English Learners (ELs) have become the fastest growing school age population in the United States. According to the National Center for Education Statistics (NCES) (2023), currently, 12 states, mainly in the Western region, along with the District of Columbia, had over 10% of their public-school students classified as ELs: notably, Texas (20.1%), California (17.7%), and New Mexico (16.0%). Additionally, 20 states had EL populations ranging from 6 to 10%, 13 states ranged from 3 to 6%, and only five states had less than 3% EL students.

When students enter school from homes where a language other than English is spoken and they are assessed to be English Learners (ELs), they are assigned to a supplemental English assistance program with the goal of improving their English language proficiency. The goal is to learn English while also gaining grade appropriate academic content competency. As students gain proficiency in English, they are assessed to determine whether they can be reclassified English proficient. Reclassification is the process where a student exhibits language proficiency adequate for academic success (as determined by the state and local education agencies) and serves as a crucial milestone for ELs to transition into English-only instructional settings.

The timing for reclassifying English Learners (ELs), however, varies significantly, ranging from short-term to long-term durations, depending on various criteria set by different states and school districts. Additionally, much of the research literature on ELs concentrates on the criteria for reclassification generally and for ensuring that ELs are reclassified at the optimal time rather than too early or too late (Betts et al., 2020; Slama et al., 2017; Shin, 2018). Long-term reclassification, specifically, has emerged as a significant concern for policymakers and educators (Haas et al., 2014; Luna, 2020; Olsen, 2014), especially in populous and high growth states like California where about one in 10 students is classified as an LTEL^1^ [California Department of Education (CDE), 2024a]. The policy and research focus on this group of students is well founded since students who are LTELs generally perform less well academically than students who meet the criteria for reclassification earlier and have more limited access to core academic content (Estrada and Wang, 2018; Umansky, 2016).

Meanwhile, in terms of *earlier* reclassification, much of the debate has been around the academic preparedness of English learner students (Betts et al., 2019). A key concern among experts and educators, for example, is that students who exit the EL category early may still not be ready for English-only instruction without some form of continued support (Thompson, 2017; Hakuta et al., 2000). Betts et al. (2020) in a study of the reclassification practices of two school districts at two different time periods cautioned that too early a reclassification could have negative ramifications for some students while too late a reclassification could likewise have a detrimental consequence for students. Nevertheless, for the majority of E-RFEPs it is possible that there are no negative academic ramifications, and in fact exiting the EL category earlier may be advantageous. There is a substantial body of research literature (Bialystok and Craik, 2010; Engel de Abreu et al., 2012; Marian and Shook, 2012; Nicolay and Poncelet, 2015) that points to both cognitive and linguistic advantages of childhood bilinguals with presumably many qualifying as E-RFEPs. For this reason, timing of reclassification is an important consideration because the criterion for reclassification can be confounded with factors such as differential ability to learn a second or even a third language, quality of second language input, time to practice a second language and motivation to learn a second language (Padilla et al., 2020). Furthermore, there may be other factors that influence the timing of reclassification. As EL students come from linguistically, socially and culturally diverse backgrounds [Government Accountability Office (GAO), 2024; Batalova and Zong, 2016; Halle et al., 2012], it is important to also examine the demographic and socioeconomic related factors that could play an important role in the timing of reclassification. However, there are no systematic longitudinal studies tracking E-RFEP students to determine their long-term academic trajectories compared to similar groups of students who take 2 or 3 or more years more to achieve reclassification or who remain as LTEL for longer periods. Instead, many studies examining longitudinal reclassification patterns focus on time to reclassification (Thompson, 2017), the effect of varying reclassification criteria on time to exiting the EL category (Cimpian et al., 2017), or the effects of reclassification during critical transition periods (Johnson, 2019) with notable exceptions described above (e.g., Betts et al., 2020; Betts et al., 2019). Accordingly, this study focuses on the differentiation of reclassification for ELs, especially focusing on E-RFEP. We ask a more nuanced set of critical policy questions, such as: What demographic or socioeconomic factors contribute to the *early* reclassification of students? And, does being an E-RFEP student have positive impacts on academic performance when compared with late reclassification (LTEL)?

## Research aims

Our goal in this investigation was to better understand the profile of Early-Reclassified Fluent English Proficient (E-RFEP) students and their longer-term academic progress and outcomes through middle and high school, as well as to compare educational outcomes with ELs who are reclassified later in school. All students included in this study had been in the U. S. educational system for the duration of their K-12 academic careers. In this study, E-RFEP refers to students reclassified as early as grades K-2 or 3–4. Three research questions are examined:

1. What is the grade level distribution for when EL students are reclassified as fluent English Proficient (RFEP)?
2. Are there demographic (parent education, students’ gender) and socioeconomic status (school lunch) that contribute to early reclassification from EL status to reclassified fluent English Proficient (RFEP)?
3. Does early reclassification (E-RFEP) contribute positively to academic achievement when compared to similar students who are on a slower trajectory of reclassification (e.g., LTEL)?

## Research methods

### Sample and procedures

This study was intended to fill the gap in our understanding of educational outcomes between early- and long-term English Learners. Among five districts working in partnership with the Stanford Graduate School of Education via a research practice partnership (RPP)^2^, we identified two school districts serving grades kindergarten through 8th grade (e.g., ages 5–14) in the RPP that had large enrollments of EL students and students on free or reduced lunch programs, therefore these two school districts were included in this study. We also identified a third school district serving grades kindergarten through 8th grade with a sufficient number of EL students, so this district was also included in the current study. Students from these three districts all transitioned to the same high school district that is also part of the partnership. Two other elementary districts in the RPP were excluded from the study because they had few EL students and limited socioeconomic variability (e.g., a small percent of students on free/reduced lunch plans). Data files for students exiting elementary 5th grade in our 3 school districts who also transitioned to middle schools (6th grade) in their respective districts and subsequently to the feeder high school district (9th grade through 12th grade) in the RPP were included for analyses in this study. Two cohorts of students who entered kindergarten in 2008–09 and 2009–10 in our target school districts were selected for this study (they were seniors in high school during the 2020–21 and 2021–22 academic years). We used the district’s initial language classification for all of the students as the starting point for the study. Students in the two cohorts were classified as: English Only (EO), English Learner (EL), or Initial Fully English Proficient (IFEP). The latter designation is used when a child is assessed proficient in English the first time they are evaluated in kindergarten, even though there was a non-English language spoken in the home. Then we tracked all ELs by the grade level year in which they were later reclassified by their respective school district as Fully English Proficient (RFEP). This allowed us to further categorize students as early, mid, or late term English Learners. Importantly, for this study our focal group of interest was Early Reclassified Fluent English Proficient (E-RFEP) students.

In total, there were 1,152 students who were initially classified as ELs when they entered kindergarten and who were later reclassified as RFEP and remained in the school systems for the duration of their K-12 educational trajectories. We included all EL students who met these criteria in the study. These students were divided between the three districts, with 996 coming from one district, 110 from another, and 46 from the third. Students came from a total of 19 different elementary schools, across the three districts. Although our primary questions of interest concern differences between EL students based on grade level year of reclassification, we also included a comparison group of 275 English Only (EO) students in order to see how each EL group compared to students who were not English Learners. We selected 275 EO students as this was approximately the size of one of our reclassification groups; the reclassification groups are explained below. The EO group was randomly sampled from the group of all EO students for whom we have data, but the sample was specified to match the EL student group distribution in terms of district and ethnicity. Therefore, 226 EO students were drawn from the district with the most ELs, and 27 and 22 from the two districts with smaller numbers of ELs. In addition, 80% of the EO students identified as Hispanic, in order to match the demographics of the EL students. More details about students’ ethnicity are reported in Research Question 2.

### Variables of interest

Data consisted of both demographic and socioeconomic variables (e.g., gender, ethnicity/race, eligible for free/reduced lunch plan-yes/no), as well as academic outcome variables—English Language Arts (ELA) and Math scores on California mandated standardized achievement tests (SBAS-ELA/MATH)^3^ for students from elementary school (grade 2) through grade 11 in high school (approximately age 7 to age 17). If students were missing data for a particular measure, they were excluded from the analysis for that measure.

### Analytical plans

As mentioned, we followed two cohort configurations beginning with kindergarten records for all entering kindergarten students with particular interest in students classified as ELs. This design allowed us to track the academic performance of students from kindergarten through 12th grade.

With the above-mentioned data, our analyses were conducted related to each of the research questions:

First, we address Research Question 1 by examining the grade level when *EL students were Reclassified Fully English Proficient (RFEP) by the district?*

Second, we address Research Question 2 by investigating *whether certain demographic and socioeconomic factors contribute to early reclassification from EL status to reclassified fluent English Proficient (RFEP)?*

Third, we address Research Question 3 by examining *whether early reclassification is associated with higher levels of academic achievement when compared to EL students who are on a late or slower trajectory of reclassification (e.g., LTEL) as well as their English-only peers?*

## Results

Here are the findings corresponding to our three research questions related to the grade levels of reclassification, factors related to E-RFEP, and academic outcomes of E-RFEP compared with LTEL and English-only (EO) peers. All data analysis was carried out in R (R Core Team, 2024).

*At what grade levels are EL students reclassified to fully English Proficient status (RFEP)?*

To address this question, we searched district records to identify the grade when students were classified as RFEP. We found wide variation, with some students being reclassified at the end of kindergarten and a few students not reclassified until 12th grade. Table 1 shows the distribution and percentage of students reclassified at each grade level.

**Table 1:** Distribution and percentage of EL students reclassified at each grade level.

Grade reclassified	Number	Percent
K	134	11.6%
1	2	0.2%
2	105	9.1%
3	203	17.6%
4	131	11.4%
**5**	**243**	**21.1%**
6	58	5.0%
7	38	3.3%
8	38	3.3%
**9**	**155**	**13.5%**
10	21	1.8%
11	12	1.0%
12	12	1.0%
Total	1,152	

Bolded rows indicate transitional points at which disproportionately large numbers of students are reclassified.

Table 1 indicates that there are certain transitional points when more students are reclassified. These reclassification points mark important transitions in educational trajectories: at the end of 5th grade and before students transition to middle school; and at the beginning of 9th grade when students enter high school. Based on the distribution of reclassification, we divided EL students into four groups based on the grades they were reclassified: grades K-2, 3–4, 5–7, and 8–12. Each group contains between 21 to 30% of the ELs in our study. Half of the students (50.9%) were reclassified in K-2 or 3–4, which we designate as E-RFEP. Table 2 provides the number of students in each of these four reclassification groups.

**Table 2:** Number and percentage of EL students in each reclassification group.

Reclassification group (Years to Reclassification)	Number	Percent
K-2 [1–3 years EL status]	241	20.9%
3–4 [4–5 years EL status]	334	30.0%
5–7 [6–8 years EL status]	339	29.4%
8–12 [9–13 years EL status]	238	20.7%
Total	1,152	

### Research question 1 summary

These results indicate that there is wide variation when EL students are reclassified as RFEP, but there are notable times of reclassification during important transition years (5th grade; 8th grade). For the purpose of analyzing reclassification, we divided ELs into four similarly sized groups based on when they were reclassified (K-2, 3–4, 5–7, 8, and above), which allowed us to examine broad patterns relating to grade of reclassification in the sections that follow.

*Research Question 2: Are there demographic and socioeconomic factors that contribute to early reclassification from EL status to Reclassified Fully English Proficient (RFEP)?*

We first report descriptive information about demographic factors for the four EL reclassification groups, as well as for an EO comparison group. Specifically, the demographic variables investigated were parent education level, lunch status, ethnicity, and gender. We chose these variables as they were the ones for which we consistently had data across our sample.

#### Parent education level

**Figure 1:**
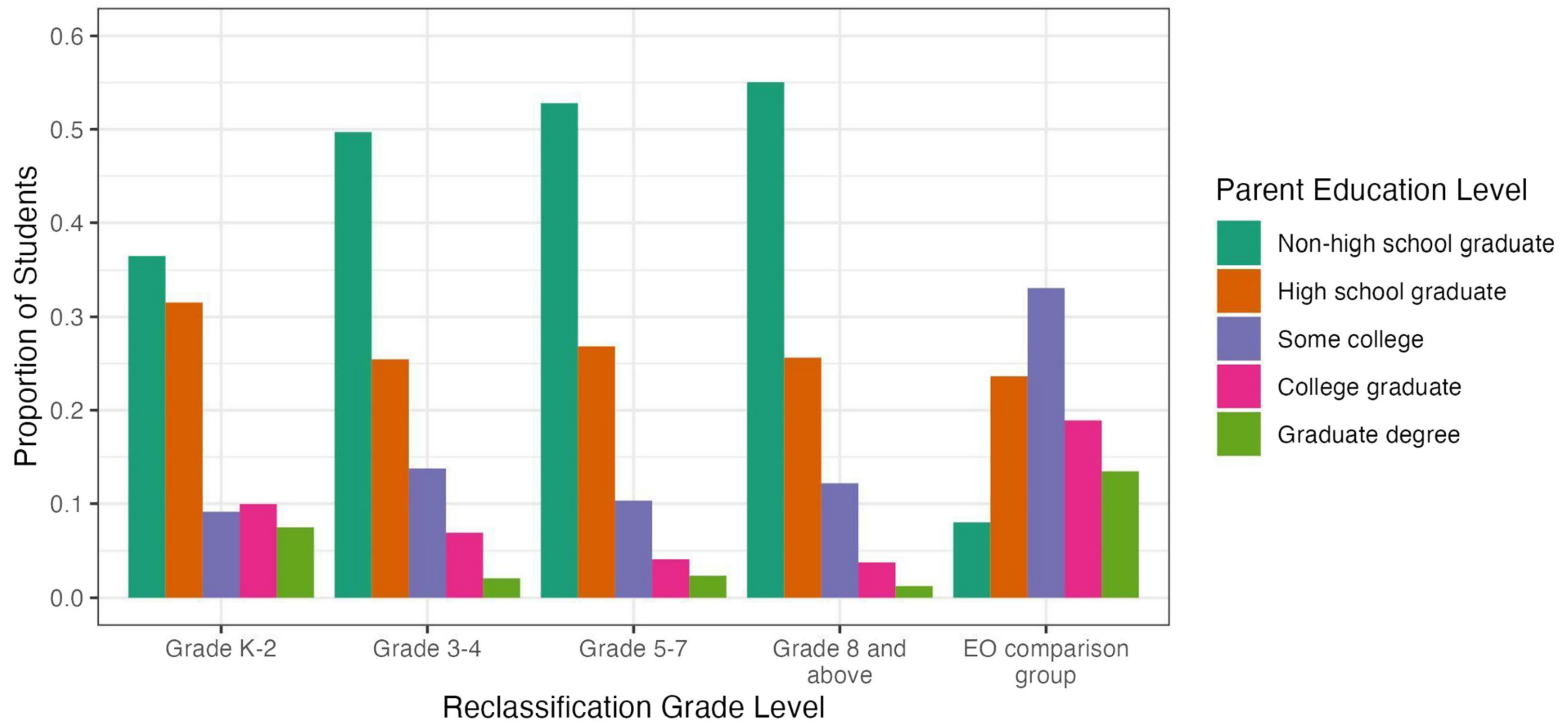
Proportion of students per parent education level for EL reclassification groups and EO comparison group.

Figure 1 provides information on the highest education level attained by either parent for students in each of the EL reclassification groups and the EO comparison group. Here, we found an overall difference between students who were reclassified early, in grades K-2, and students who were reclassified in grades 3–4, 5–7, or 8–12; the latter three groups all had similar profiles to one another. Students reclassified in grades K-2 were less likely (37%) than later-reclassified students (>49% for each group) to have a parent who was a non-high school graduate. Early-reclassified students were also somewhat more likely to have a parent who was a high school graduate (32%, vs. 20–25% for later-reclassified students) or had a college degree or an advanced graduate degree (18%, vs. 5% or less for later-reclassified students).

Compared to the EL groups, students in the EO comparison group were less likely to have a parent who was not a high school graduate (7%) and more likely to have a parent with some college (33%) or with a college degree (19%) or an advanced graduate degree (13%).

Thus, students who were reclassified early (grades K-2) had parents who on average had more education than parents of students who were reclassified later.

#### Lunch status

**Figure 2:**
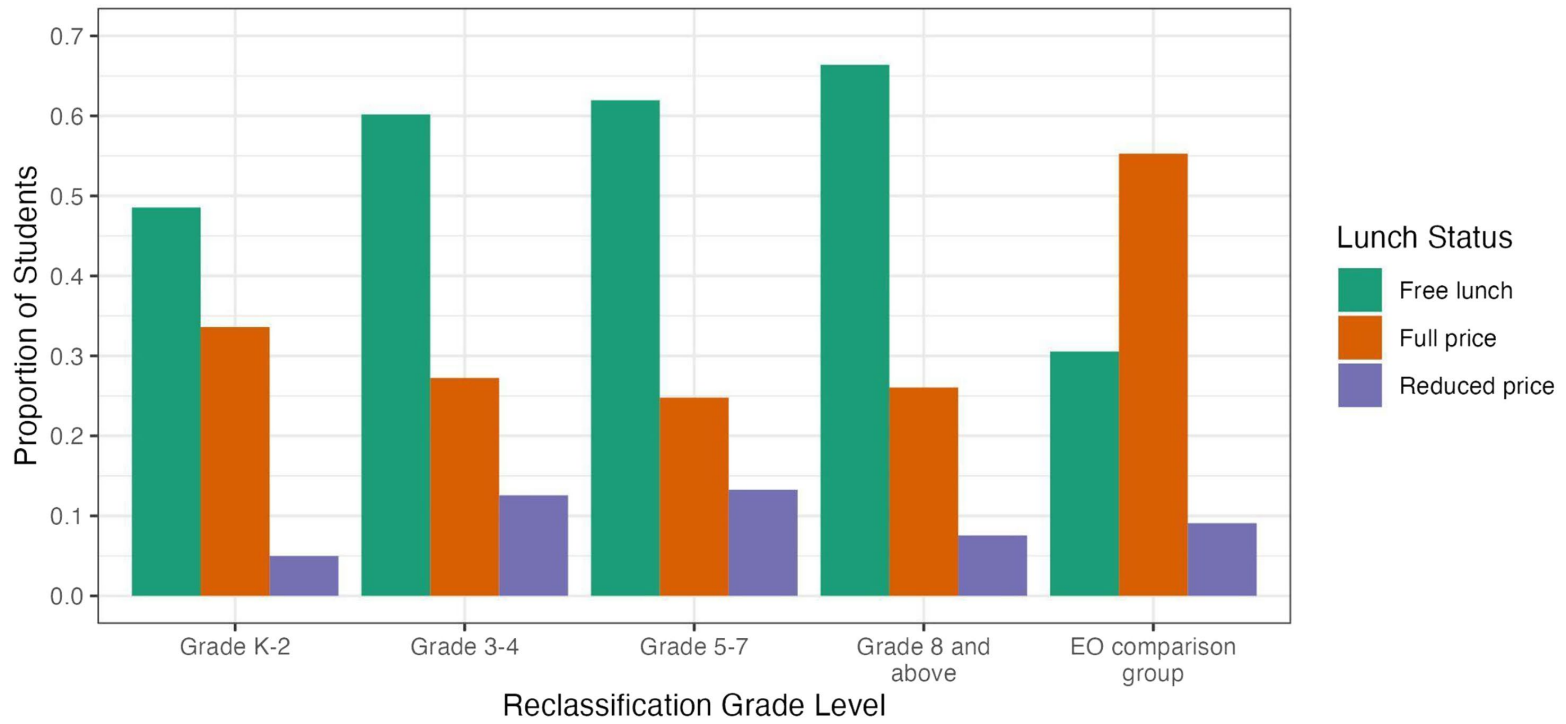
Proportion of students per lunch status for EL reclassification groups and EO comparison group.

Figure 2 reports the lunch status (free lunch, full price, or reduced price) for the EL reclassification groups and the EO comparison group. EL students reclassified in grades K-2 were somewhat less likely to be eligible for free lunch (49%), and more likely to pay full price (33%), with few (about 5%) qualifying for reduced price. Students reclassified in grades 3–4, 5–7, or 8 and above had a similar lunch status profile to one another: 60–70% qualified for free lunch, about 25% paid full price, and 10–15% qualified for reduced price.

In the EO comparison group, students were less likely than the EL students to qualify for free lunch (31%) and more likely to pay full price (55%).

The lunch status data indicates that students who were reclassified early in grades K-2 had parents who were higher in socioeconomic status (SES) and were less likely to qualify for free or reduced lunch—than families whose students were reclassified as English proficient later in school.

#### Ethnicity

The majority of students in each group identified as Hispanic, with fewer students identifying as Hispanic in the early exit K-2 group (59%) compared to the other groups (all >80%). 7% of students in the early K-2 exit group identified as White, 5% as Asian, and 7% as Pacific Islander. In addition, 80% of the EO comparison group identified as Hispanic, 10% White, and the remaining 10% consisted of students of Asian, Pacific Island, and other ethnic origin backgrounds.

Overall, students who were reclassified later in grades 3–4, 5–7, or 8 and above were more likely to identify as Hispanic (approximately 90%) than students who were reclassified early in grades K-2.

#### Gender

For students reclassified early in grades K-2 or 3–4, and for students in the comparison group, about 55% identified as female and about 45% as male. For students who were reclassified later in grades 5–6 or 8 and above, about 55% identified as male and about 45% as female. In sum, students who were reclassified earlier were slightly more likely to be female than male in our sample.

#### Which demographic factors predict early reclassification?

To determine which demographic factors significantly predicted early reclassification among EL students, we created a linear regression model with the following predictors: highest parent education level, lunch status, ethnicity, and gender. The outcome variable was students’ grade of reclassification (the exact grade, not the reclassification group, e.g., grade 3). These predictor variables were those for which we had the most consistent data, and for all of them we found indications of differences between reclassification groups in our descriptive results summarized above. The results of the regression analysis are shown in Table 3.

**Table 3:** Results of linear regression model predicting students’ grade of reclassification.

Coefficients	Estimate	Std. Error	*t*-value	*p-*value
Intercept	3.49	0.60	5.79	<0.001***
Gender: male	0.49	0.17	2.87	<0.01**
Parent education level: Graduate degree	−0.57	0.61	−0.92	0.36
Parent education level: College graduate	−0.62	0.40	−1.56	0.12
Parent education level: Some college	−0.05	0.28	−0.19	0.85
Parent education level: High school graduate	−0.38	0.20	−1.90	0.06 ^+^
Parent education level: Decline to state	−0.79	0.49	−1.62	0.11
Lunch status: Full price	−0.14	0.21	−0.68	0.50
Lunch status: Reduced price	−0.09	0.28	−0.33	0.74
Ethnicity: Asian	−0.58	0.85	−0.68	0.50
Ethnicity: Black	−2.70	2.86	−0.95	0.34
Ethnicity: Hispanic	1.50	0.58	2.59	<0.01**
Ethnicity: Multiple	−3.12	2.83	−1.10	0.27
Ethnicity: Native American	−0.29	1.36	−0.21	0.93
Ethnicity: Pacific Islander	0.05	0.76	0.07	0.95
Ethnicity: Unknown	−0.17	0.67	−0.25	0.80

Significance codes: ***p < 0.001; **p < 0.01; *p < 0.05; ^+^*p* < 0.1. R^2^ = 0.07; adjusted R^2^ = 0.06.

The regression analysis found that male students were reclassified significantly later than female students (*p* < 0.01) and that Hispanic students were reclassified significantly later than non-Hispanic students (*p* < 0.01). In addition, students with one or both parents who were high school graduates were reclassified earlier than students whose parents were not high school graduates, reaching marginal significance (*p* = 0.06). However, the relatively low R^2^ value (0.07; adjusted R^2^ = 0.06) suggests that there is still a large amount of variance left unexplained.

### Research question 2 summary

In sum, the descriptive statistics suggest that students reclassified early in grades K-2 differed from students reclassified later in terms of level of parent education, lunch status, ethnicity, and gender. Supporting this, a linear regression model found that students whose parents were more educated, who did not identify as Hispanic, and who were female were more likely reclassified earlier than other students. However, there are likely other factors contributing to students’ age of reclassification that are not captured in the model (e.g., students with more educated parents may have been enrolled in a pre-kindergarten school readiness program where they were exposed to English and acquired some English proficiency prior to kindergarten entry). Whether this was the case was not recorded on the school record for these early EL reclassified students.

*Research Question 3: Does early reclassification contribute to academic achievement when compared to similar students on a slower trajectory of reclassification (e.g., LTEL)?*

To examine the academic achievement of students reclassified at different grade levels, we compared EL students from the different reclassification groups, as well as the EO comparison group, on the following measures: SBAC ELA scores (grades 2–8), SBAC Math scores (grades 2–8), SBAC Science scores (grades 5, 11, and 12), and high school GPA (grades 9–12). Due to interruptions in testing as districts switched to a different assessment system during the pandemic years, SBAC ELA and Math scores for students’ high school years were nonexistent. In addition, since the SBAC ELA and Math results were not on the same scale for students’ elementary and middle school test results, the achievement scores were examined separately for grades 2 to 4; and scores from grades 5 to 8.

#### SBAC ELA

**Figure 3:**
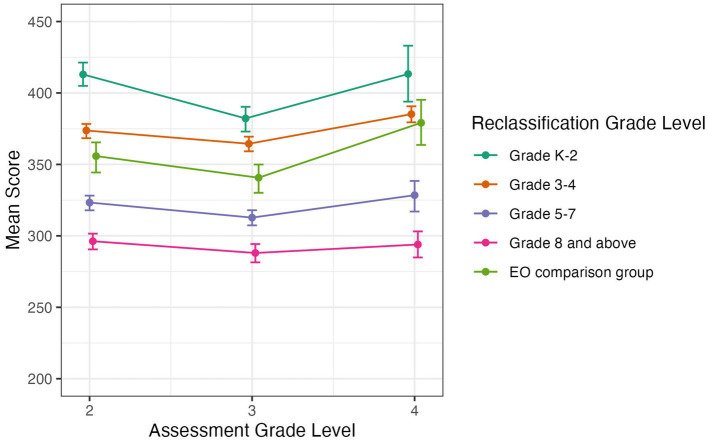
Average SBAC ELA score by grade level for students in EL reclassification groups and EO comparison group, in grades 2 to 4. Error bars represent bootstrapped 95% confidence intervals.

**Figure 4:**
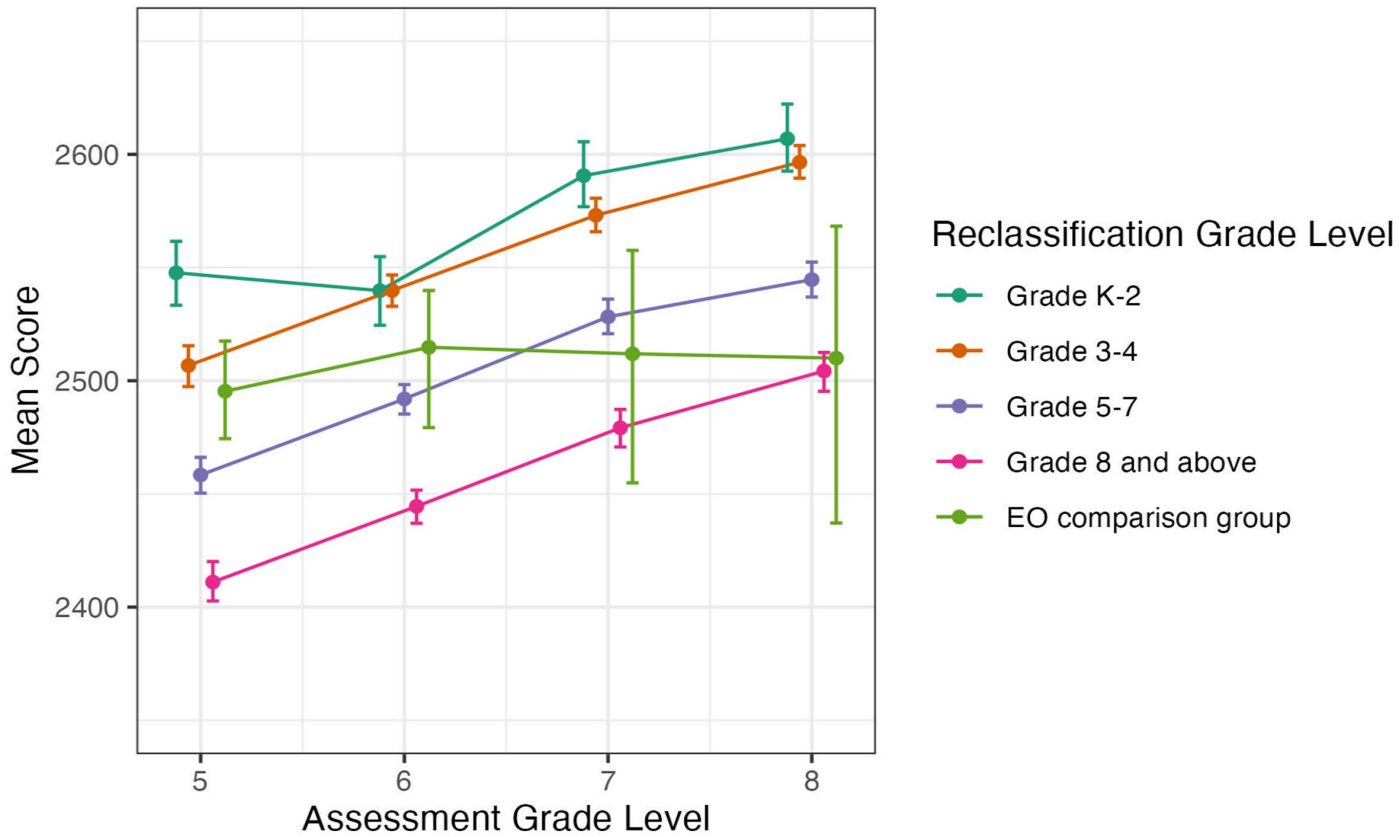
Average SBAC ELA score by grade level for students in EL reclassification groups and EO comparison group, in grades 5 to 8. Error bars represent bootstrapped 95% confidence intervals.

Figure 3 below illustrates students’ SBAC ELA scores from 2nd to 4th grade, and Figure 4 provides their SBAC ELA scores from 5th to 8th grade. Error bars represent 95% confidence intervals. Inspection of the figures show that students reclassified early in grades K-2 or grades 3–4 had consistently higher SBAC ELA scores than students reclassified later in grades 5–7 or grade 8 and later in high school. Also, students reclassified in grades K-2 or grades 3–4 had similar to or higher SBAC ELA scores than students from the EO comparison group.

To test for differences in SBAC ELA scores between groups, we carried out post-hoc Tukey’s Honestly Significant Difference (HSD) tests (Tukey, 1949). In grades 2 and 3, there were significant differences between all groups (all *p* < 0.05, and with mostly *p* < 0.01), with students reclassified early in grades K-2 having higher test scores, followed by students reclassified in grades 3–4, then the EO comparison group, followed by students reclassified in grades 5–7, and lastly students reclassified in grades 8 or later having the lowest test scores (Figure 3). The same pattern held for students reclassified in 3rd–4th grade except that the difference between students reclassified in grades K-2 and grades 3–4 was marginal (*p* = 0.06), and there was not a significant difference between students reclassified in grades 3–4 and the EO comparison group.

In grades 5 to 8, students reclassified in grades K-2 or in grades 3–4 had significantly higher SBAC ELA scores than students reclassified later in grades 5–7 or grade 8 and above (all *p* < 0.01; Figure 4). In 5th grade, students reclassified in grades K-2 also had significantly higher scores than students reclassified in grades 3–4 (*p* < 0.01). In addition, from 5th through 7th grade, students reclassified in grades 5–7 had significantly higher SBAC ELA scores than students reclassified in grade 8 or later (*p* < 0.01), and there was not a significant difference for students reclassified in the 8th grade or later. Because the scores in the EO comparison group were quite variable, especially in grades 7 and 8, there were no clear patterns in the tests for that group.

In sum, students who were reclassified earlier had higher average SBAC ELA scores, with the clearest pattern for students reclassified in grades K-2 or in grades 3–4 earning higher scores on the SBAC ELA than students reclassified in later grades (i.e., 5th through 9th grade).

#### SBAC math

**Figure 5:**
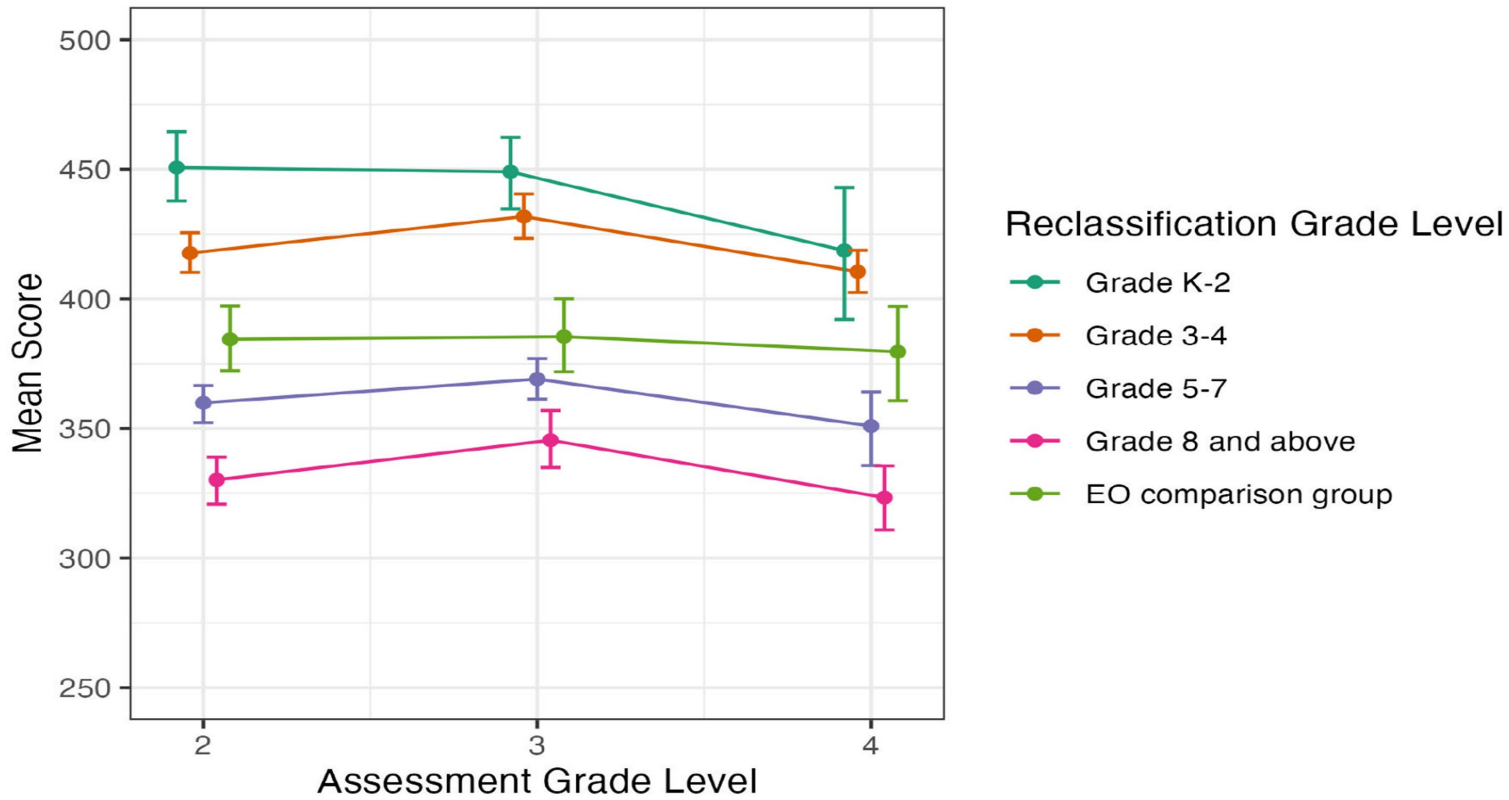
Average SBAC Math score by grade level for students in EL reclassification groups and EO comparison group, in grades 2 to 4. Error bars represent bootstrapped 95% confidence intervals.

**Figure 6:**
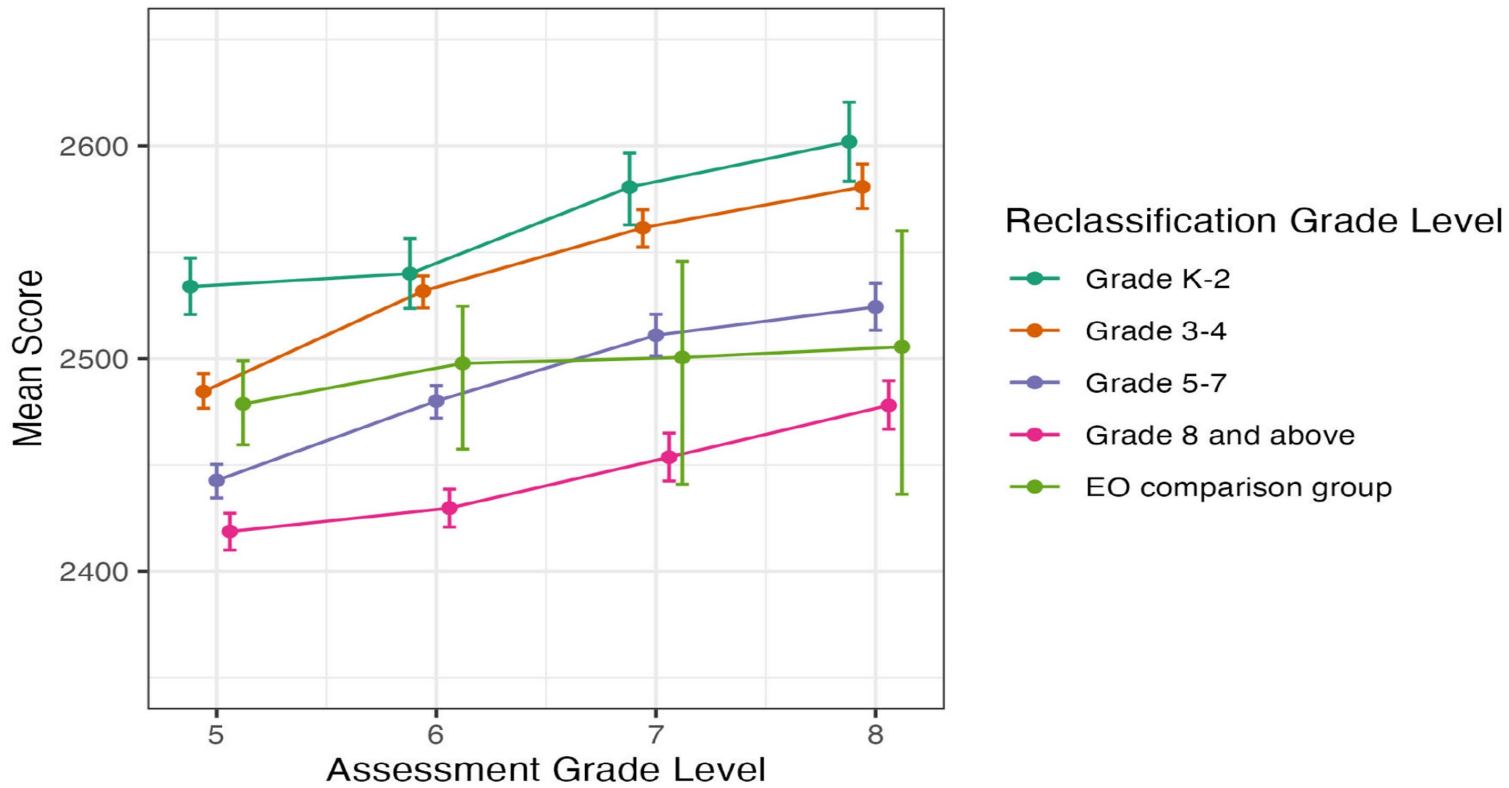
Average SBAC Math score by grade level for students in EL reclassification groups and EO comparison group, in grades 5 to 8. Error bars represent bootstrapped 95% confidence intervals.

Figures 5, 6 show students’ SBAC Math scores by reclassification group for grades 2–4 and grades 5–8, respectively. Similar to the SBAC ELA scores, the data show that students reclassified earlier in grades K-2 or grades 3–4 had consistently higher SBAC Math scores than students reclassified later in grades 5–8 or in high school. Early-reclassified students also had similar to or higher Math scores than the EO comparison group.

Using Tukey’s HSD tests, we found that in grades 2–4, most of the comparisons between groups were significant (*p* < 0.05, with most < 0.01). The overall pattern was that students reclassified early in grades K-2 generally had higher SBAC Math scores, followed by students reclassified in grades 3–4, than the EO comparison group, followed by students reclassified in grades 5–7, and finally those reclassified in grade 8 and above (Figure 5). However, in 3rd and 4th grade, there was not a significant difference between students reclassified in grades K-2 and 3–4. Also, in 3rd grade there was not a significant difference between the EO comparison group and students reclassified in grades 5–7, and the difference between the EO comparison group and students reclassified in grades K-2 was marginal (*p* = 0.06).

In grades 5–8, students who were reclassified in grades K-2 or grades 3–4 had significantly higher scores than students reclassified in grades 5–7 or grade 8 and above (all *p* < 0.01). Also, students reclassified in grades 5–7 had significantly higher scores than students reclassified later in grade 8 and above (all *p* < 0.05). In 5th grade, specifically, students from the K-2 group had scores that were significantly higher than students from the 3–4 group (*p* < 0.01). Again, the scores of the students in the EO comparison group were variable and did not show a clear pattern in the significance tests (Figure 6).

#### SBA science

**Figure 7:**
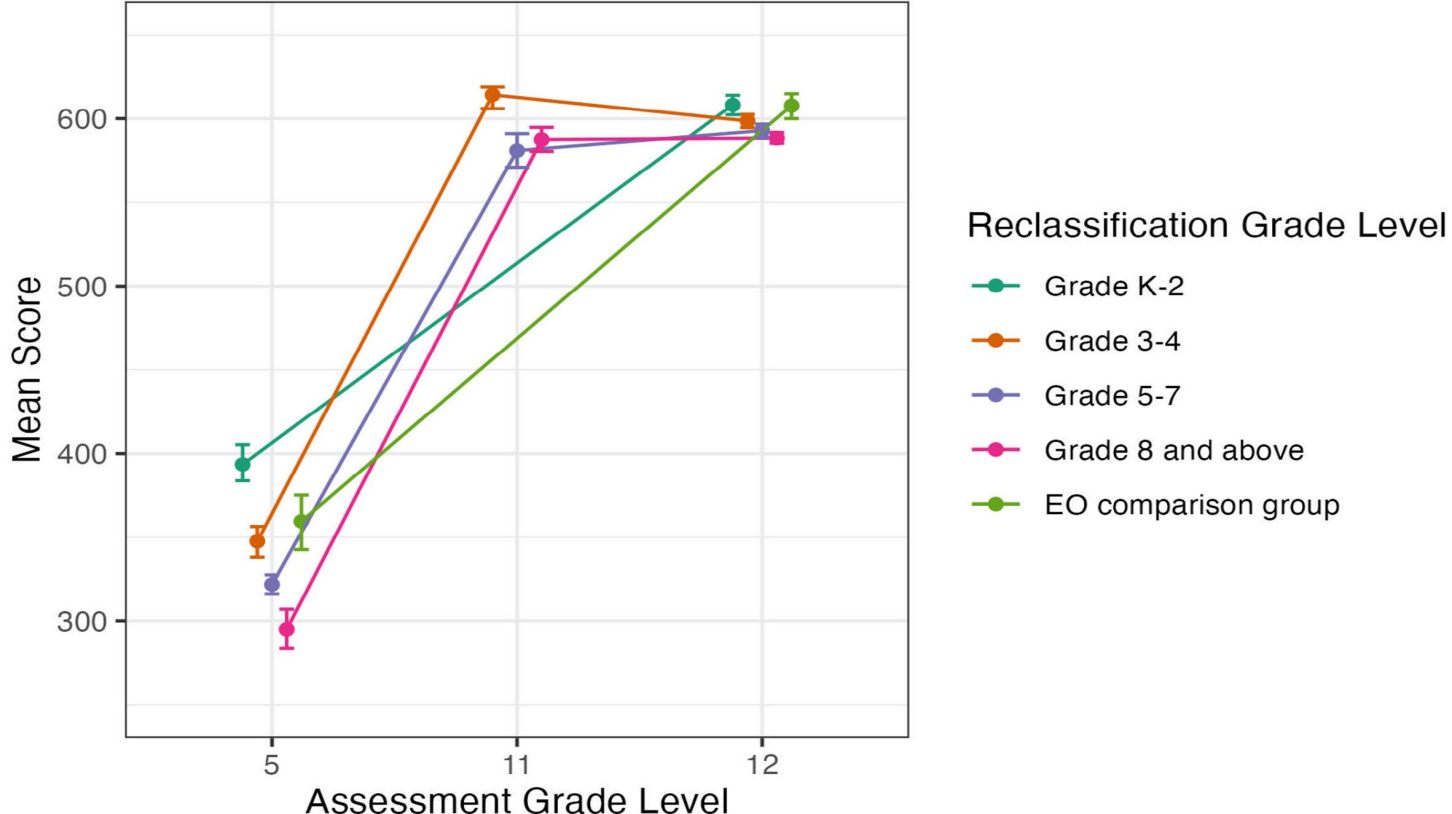
Average SBAC Science score by grade level for students in EL reclassification groups and EO comparison group, in grades 5, 11, and 12. Error bars represent bootstrapped 95% confidence intervals.

Figure 7 illustrates the mean SBAC Science score by grade level for the three grades that we have data for: 5th, 11th, and 12th grade. In 11th grade, we do not have data for students reclassified in grades 3–4 or the EO comparison group.^4^

Tukey’s HSD tests found that in 5th grade, all comparisons, but one were significant, with students who were reclassified in grades K-2 having the highest SBAC Science scores, followed by students reclassified in grades 3–4 or who were in the EO comparison group, followed by students reclassified in grades 5–7, and finally late reclassified students (grade 8 or above; all *p* < 0.01). There was no significant difference in average scores between students reclassified in grades 3–4 and the EO comparison group. On the 11th grade assessment, students who were reclassified in grades 3–4 scored significantly higher than students reclassified in grades 5–7 (*p* = 0.02) or later in grade 8 and above (*p* = 0.02), and the latter two groups did not differ significantly from each other.

Finally, in 12th grade, students reclassified Early had significantly higher Science scores than students reclassified in grades 5–7 (*p* < 0.01) or in grade 8 or later (*p* < 0.01). Early reclassified students also had higher science scores than students reclassified in grades 3–4, with the difference reaching marginal significance (*p* = 0.06). Students reclassified in grades 3–4 scored significantly higher than students reclassified later in grade 8 or later in high school (*p* < 0.01). The EO comparison group had significantly higher scores than students reclassified in grades 5–7 (*p* < 0.01) or later (i.e., grade 8 or high school; *p* < 0.01). Other comparisons were not significant.

Overall, for SBAC Science (Figure 7), there is again a general pattern where students reclassified Early—particularly in grades K-2 and grades 3–4 show higher Science scores than students reclassified Late, particularly in grades 5–8 or in high school.

#### High school GPA

**Figure 8:**
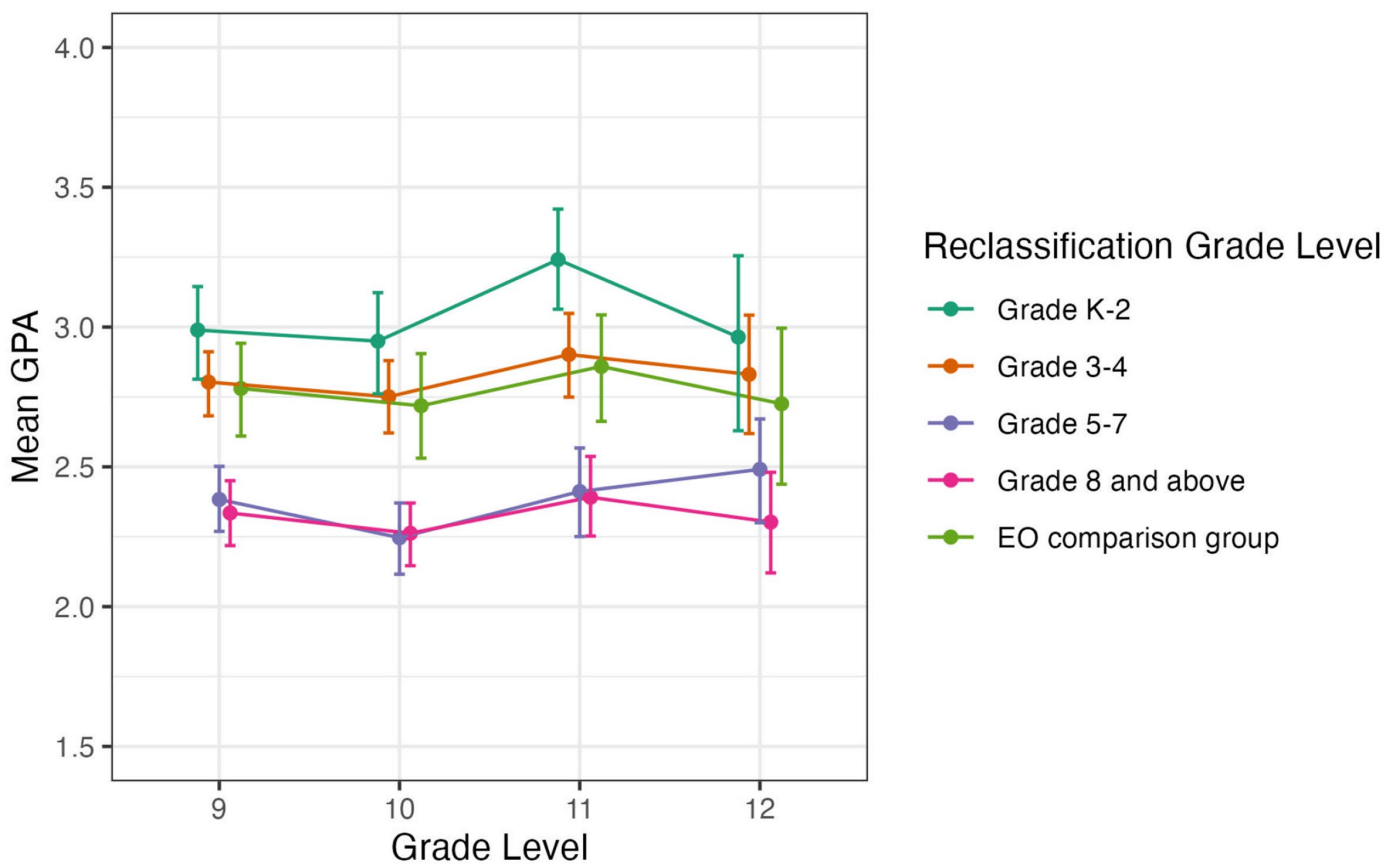
Average high school GPA by grade level for students in EL reclassification groups and EO comparison group. Error bars represent bootstrapped 95% confidence intervals.

Figure 8 shows the mean GPA by grade level (95% confidence intervals) for students from each reclassification group and the EO comparison group. For each grade level in high school (9–12), the graph shows that students reclassified Early in grades K-2 and grades 3–4 had similar average GPAs as students from the EO comparison group. Students who were reclassified in grades 5–7 or grade 8 and above had a lower average GPA.

Tukey’s HSD tests were used to test for significant differences in grade level GPAs between groups. For 9th and 10th grade, the pattern of results was the same: Students reclassified in grades K-2 or in grades 3–4 had significantly higher GPAs than students reclassified in grades 5–7 (all *p* < 0.01) or in grade 8 and above (all *p* < 0.01). The EO comparison group also had a significantly higher mean GPA than students reclassified in grades 5–7 (both *p* < 0.01) or grades 8 and above (both *p* < 0.01), but there was no a significant difference in GPA between the EO comparison group and students reclassified in grades K-2 or in grades 3–4. For 11th grade GPA, students reclassified Early again had a significantly higher mean GPA than students reclassified in grades 5–7 (*p* < 0.01) or grades 8 and above (*p* < 0.01). They also had a significantly higher GPA than the EO comparison group (*p* < 0.05) and a higher GPA than students reclassified in grades 3–4, reaching marginal significance (*p* = 0.06). Students reclassified in grades 3–4 had a significantly higher mean GPA than students reclassified in grades 5–7 (*p* < 0.01) or in grade 8 and above (*p* < 0.01). The EO comparison group had a significantly higher GPA than students reclassified in grades 5–7 (*p* < 0.01) or grade 8 and above (*p* < 0.01). There was not a significant GPA difference between students reclassified in grades 5–7 and students reclassified in grade 8 and above.

For 12th grade GPA, students reclassified Early in grades K-2 had a significantly higher GPA than students reclassified Later in grade 8 and above (*p* < 0.01), and so did students reclassified in grades 3–4 (*p* < 0.01). No other comparisons were significant.

This points to an overall pattern where students who were reclassified Early in grades K-2 or in grades 3–4, as well as students from the EO comparison group, showed a higher overall high school GPA than students who were reclassified Later. Thus, students who are reclassified Early show higher academic achievement levels on standardized measures of achievement as well as GPAs than similar EL students who were reclassified later in middle school or in high school.

### Research question 3 summary

Across SBAC ELA, Math, and Science scores, as well as high school GPA, the results showed that students reclassified Early (grades K-2 or 3–4) had higher academic achievement scores than students who were reclassified later (grades 5–7 or 8 and above). Also noteworthy is that Early-reclassified students often had achievement scores that were similar to or higher than those of the EO comparison group.

## Discussion

For the first research question: *At what grade levels are EL students reclassified to Fully English Proficient status (RFEP)?* Reclassification is a multifaceted process and is an important milestone in ELs academic development and it varies by reclassification procedures used in different locales and states (Johnson and Goldenberg, 2020). Results presented in this study showed that although there are some indicators for when students are more likely to be reclassified (e.g., transition from K to 1st grade, end of elementary school, end of middle school, high school entrance), there’s considerable variability in the timing of when ELs transition to Reclassified Fluent English Proficient (RFEP) status with half of the EL students (49.9%) reclassified in K-2 or grades 3–4, which we here label as Early-RFEP. We categorized EL students into four groups of approximately similar size according to time to reclassification: K-2, 3–4, 5–7, and 8 and above. Our results provide more detailed information about the trajectory of reclassification. In addition, the current results align with the overall pattern of previous findings that show reclassification to be completed prior to the transition from elementary and middle school (e.g., Greenberg Motamedi, 2016; Slama, 2014). Our findings also conform to a similar pattern with the peak of reclassification, occurring in 5th grade with 71% of students reclassified, whereas a report from Los Angeles Unified School District (LAUSD; Thompson, 2017) indicated the peak reclassification to occur at the end of elementary school (i.e., grade 6), and with a total of 74% of students reclassified by the end of middle school. In sum, the current study contributes to the small but growing literature using a longitudinal design to understand the timing to reclassification among ELs which offers to broaden educators’ awareness of the differentiation among EL sub-groups with varied reclassification trajectories.

In terms of the second research question: *What demographic or socioeconomic factors contribute to early reclassification from EL status to Reclassified Fully English Proficient (RFEP)?* Our results show that the predictor variables for the higher achievement among Early ELs are parental education level, free/reduced lunch status, ethnicity, and gender. Previous studies have discussed factors that contribute to the time to classification in general, such as spoken languages, language programs (Umansky and Reardon, 2014), type of English language development program (Thompson, 2017), and classroom teacher characteristics (Joshi, 2023). These programmatic factors are not examined in our study because our data did not provide relevant information to include these variables in our model thereby leaving a large amount of variation in grade of reclassification unexplained. However, the current study is important and unique, as it illustrates the demographic and socioeconomic factors that impact students’ early reclassification specifically, parental education level, ethnicity, and gender. These results are consistent with studies that have shown evidence for individual demographic and family socioeconomic background characteristics that contribute to differential reclassification patterns (Umansky et al., 2020). Specifically, in this study, students with at least one parent who completed high school and/or some college, students who did not identify as Hispanic, and female students were more likely to be reclassified earlier than their counterparts who did not share these characteristics. This finding is not surprising: students with more educated parents likely possess greater social, economic, and educational capital because these parents are able to navigate the bureaucracy of English learner reclassification and presumably provide more resources or opportunities for learning than parents with less than a high school diploma. Thus, parents’ education level may offer a facilitation effect that contributes to ELs students acquiring English earlier while also enabling their academic/content knowledge resulting in early reclassification. Secondly, non-Hispanic EL students may have parents who have completed more years of education coupled with teachers’ who possess a mindset that positively favors non-Hispanic EL students (Padilla et al., 2022). In addition, the social and cultural capital within their non-Hispanic households or communities may contribute to their faster English language proficiency and academic opportunities, leading to earlier reclassifications (Kiley, 2019; Umansky et al., 2020) in ways unexplored in this study. Finally, the gender gap in time to reclassify as fluent English proficient that favors girls may be due to several considerations. First, it is a well-documented pattern that girls outperform boys in academic settings (Fortin et al., 2015) perhaps due to being rewarded for positive classroom behavior and self-control in classroom settings (Duckworth et al., 2015). For ELs, this may also be due to both instrumental and integrative motives—doing well in school (i.e., instrumental) and learning about school and American culture faster (i.e., integrative) and having English speaking friends (Denies et al., 2022) may be a greater motivation among girls than boys. Another consideration is that since most elementary school teachers are women, English speaking female teachers may be more impactful as language teachers and role models on girls than boys in the classroom (Denies et al., 2022). Girls may also use more effective second language learning strategies and consequently have greater success earlier as English learners and be more motivated to learn than their male counterparts, resulting in more positive learning gains on achievement tests and grades (Van de Gaer et al., 2009), culminating in earlier reclassification as E-REFP. We speculate that the downside for males with the gender gap in earlier reclassification of females is that boys possibly fall further behind academically because of longer periods spent in classes for English learners rather than in content-based classes necessary for successful academic advancement from elementary through high school.

For the third research question: *Does early reclassification contribute to academic achievement when compared to similar students who are on a slower trajectory of reclassification (e.g., LTEL)?* The analysis of SBAC ELA, Math, and Science scores, along with high school GPA, revealed that students reclassified early (grades K-2 or 3–4) generally achieved higher scores compared to Late reclassified ELs (grades 5–7 or 8 and above). Surprisedly, the Early-reclassified students often attained scores comparable to or even surpassed the achievement levels of the English Only (EO) comparison group. These findings demonstrate that the academic benefits of the Early Reclassified Fluent English Proficient [E-RFEP] group are long lasting and persist through middle and high school. Students reclassified earlier consistently outperformed their late exit counterparts in terms of varying measures of academic achievement.

## Implications

It is a well-known fact in the field of applied linguistics that learning a second language does not follow a single trajectory for learners (Parkinson and Dinsmore, 2019; Sussman et al., 2023). A large number of variables can affect both the speed at which a new language is acquired and the ultimate level of proficiency that is attained in the second language. Much of the knowledge that is known about the process of learning a second language seems not to have crossed into the field of educational assessment of English Learners. This current study helps us to better understand the reclassification of ELs as a spectrum, from early to later reclassification, the demographic and socioeconomic factors that contribute to E-RFEP, and the academic impacts of E-RFEP when compared with LTELs. Our findings can be used to help language and education researchers, school practitioners, and policymakers to reconceptualize different rates/status of reclassification, which may have further implications for assessment practices of EL students. Practical implications might include a better understanding of the negative consequences of reclassification procedures that keep EL students behind in content knowledge while holding them in an English language acquisition program that proves detrimental resulting in long term ELs languishing in grade level content knowledge while striving to improve their English proficiency. There needs to be a redirection in the concern for LTELs that is more focused on increasing educational opportunities and resources for EL (e.g., teaching strategies, curriculum design and reclassification practices), that better reflect policies to support the complexities of language learning and earlier reclassification of ELs.

## Limitation

Several limitations need to be considered. First, the participants in our current study involved only two specific cohorts from school districts involved in a Research Practice Partner (RPP) in the San Francisco Bay area and findings may not generalize to other locales. Therefore, future research is encouraged to include a more diverse population of school districts with more diverse socioeconomic backgrounds. Second, the data collected for analyses consist only of student data; however, many other factors could contribute to early reclassification. This was evident given that our model explained a relatively low amount of the variation in age of reclassification. Therefore, future studies should include other demographic and school characteristics, such as parental and teacher input, to triangulate instructional practices that take advantage of a students’ linguistic assets rather than deficits for the purpose of boosting academic self-competence and learning. Third, this study only assesses the academic impacts of early reclassification. It would be important for future studies to investigate other non-academic aspects, such as social–emotional learning and well-being, which some researchers have already begun to do (Lee and Soland, 2023).

## Conclusion

The findings of this current longitudinal study contribute to the literature in two ways: First, instead of comparing the academic outcomes of reclassified and non-reclassified students in two big groups (e.g., Slama et al., 2017), this study takes a deeper look at the comparisons among four subgroups based on the time in school (grade levels) and the time needed for ELs to be reclassified as fluent English proficient, as well as the inclusion of a comparison group of EO students from the same 3 elementary school districts matched for ethnicity, gender, parent education level and free/reduced lunch status. This provides us with a more holistic and nuanced picture of the academic impacts of reclassification. Second, our findings fill the gap in the unbalanced work that is heavily focused on academic languishing issues identified with those ELs who take longer to be reclassified (i.e., LTEL), but which ignore the academic flourishing noted with Early exit students (Clark-Gareca et al., 2020; Menken and Kleyn, 2010; Kim, 2017).

## Data Availability

The datasets presented in this article are not readily available because privacy. Requests to access the datasets should be directed to apadilla@stanford.edu.

## Footnotes

^1^California is one of the only states in the country that has specific delineations for which students are considered long term English learners [e.g., students who have been in the U.S. education system for six or more years and have remained in the same English language proficiency level for two or more years (California Department of Education (CDE), 2024b)].

^2^RPPs are “long-term, systematic efforts to improve schools and school systems through research” (Coburn et al., 2019). As such school districts collaborate with university researchers to identify and work jointly to solve complex educational problems by sharing school data and expertise.

^3^Smarter Balanced Assessment System Test - ELA (English Language Arts) and Math; retrieved June 26, 2024; smarterbalanced.org/our-system/smarter-system.

^4^These data were not available in the data archive, nor could they be located in the school districts in the study.
